# Positively worded subscale score of the Perceived Stress Scale is associated with cognitive domain function

**DOI:** 10.4236/jbbs.2017.77023

**Published:** 2017-07-24

**Authors:** Julie M. Jiang, Elizabeth K. Seng, Molly E. Zimmerman, Mimi Kim, Richard B. Lipton

**Affiliations:** 1 Saul R. Korey Department of Neurology, Albert Einstein College of Medicine, Bronx, NY, United States; 2 Department of Epidemiology and Population Health, Albert Einstein College of Medicine, Bronx, NY, United States; 3 Ferkauf Graduate School of Psychology, Yeshiva University, Bronx, NY, United States

**Keywords:** Perceived Stress Scale, cognitive domain function, cross-sectional, Perceived Stress Scale Subscales

## Abstract

**Objectives:**

To examine the cross-sectional associations of the separate subscales of the Perceived Stress Scale (PSS) and tests measuring cognitive domains in older adults

**Methods:**

897 adults over the age of 70 free of amnestic mild cognitive impairment and dementia and enrolled in the Einstein Aging Study made up the study sample. The PSS-14 was used to measure stress. Three cognitive domains (language, episodic memory, and frontal-executive) had previously been found using principle component analysis. Linear regression analyses were used to determine the relationship between the PSS subscales and cognitive domain function.

**Results:**

The study sample had a mean age of 79.1 years and 62.8% were female. Bivariate correlations show that the PSS-14 positively worded subscale of the PSS (PSS-PW) was significantly associated with all three cognitive domains (language: r = −0.15, p < 0.001; episodic memory: r = −0.16, p < 0.001; frontal-executive: r = −0.21, p <0.001) while the negatively worded subscale of the PSS (PSS-NW) was not significantly associated with any cognitive domain. In linear regression analyses adjusted for age, white race, gender, years of education, and depressive symptoms, the PSS-PW remained significantly associated with each of the cognitive domains. The PSS-NW was not associated with any cognitive domains in any model. The PSS-14 was significantly associated with language and episodic memory, but not the frontal-executive domain.

**Conclusion:**

Worse PSS-PW scores are associated with reduced cognitive function in the executive, memory, and language domains in nondemented older adults. The PSS-PW subscale correlated better with cognitive function than the overall PSS-14. Future research should evaluate the temporality of the association and if stress reduction therapies improve cognitive performance.

## 1. Introduction

The number of adults older than 65 years old is projected to grow from 524 million worldwide in 2010 to nearly 1.5 billion in 2050 [1]. Cognitive decline has been associated with increased age [2]. Individuals vary widely in rate and extent of cognitive decline, leading to investigations into factors that may contribute to the acceleration or delay of cognitive decline [3]. Stress is associated with cognitive impairment, and could be a modifiable target for intervention [4].

A situation is perceived to be stressful if environmental demands exceed an individual’s coping resources [5]. Various measures related to stress, including life events, neuroticism, and cortisol, have been linked to cognitive decline, particularly memory decline [6–9]. Perceived stress has been shown to predict the transition from normal cognition to amnestic mild cognitive impairment (aMCI) and from aMCI to dementia [10,11]. However, little research has examined the relationship between perceived stress and domains of cognitive function in nondemented older adults. The cognitive domains that we will be examining are the language, episodic memory, and frontal-executive domains.

The Perceived Stress Scale (PSS) is a 14 item questionnaire assessing subjective stress [12]. It is made up of 7 items with positive content and 7 items with negative content. While most studies use the PSS as a single summary score, research has suggested that the two subscales measure different properties of stress [14, 15]. Factor analyses have consistently shown that the positively worded items form one factor (PSS-PW) and the negatively worded items form a second factor (PSS-NW). These two factors are poorly correlated in older adults (r = −0.28) [13]. We have validated and investigated the properties of these subscales [11]. Using data from the Einstein Aging Study (EAS), we found that only the PSS-PW subscale predicts the transition from normal cognition to amnestic mild cognitive impairment (aMCI) and from aMCI to dementia [11,16]. Some researchers have hypothesized that the PSS-PW subscale measures perceived coping ability and the PSS-NW subscale measures perceived helplessness [12,15]. In this study, we will examine the cross-sectional association of the two components of the PSS with three cognitive domains: executive function, memory, and language function.

## 2. Methods

### 2.1. Study Population

The Einstein Aging Study (EAS) is a prospective, longitudinal observational study of community residing adults over the age of 70 conducted in Bronx, NY. Detailed study design and methods can be found elsewhere [17]. In summary, participants were recruited by systematically sampling Medicare and New York City voter registration lists for Bronx County, New York from 2006–2015. Eligible participants were noninstitutionalized, ambulatory, English speaking Bronx residents. Visual or auditory impairments or psychiatric symptoms that would interfere with the ability to complete study assessments served as exclusion criteria. Additionally, for these analyses, we excluded all persons who did not complete the PSS or had a diagnosis of aMCI or dementia. Our study population consists of 897 cognitively normal participants.

### 2.2. Clinical Evaluation

In-person evaluations were completed by trained research coordinators at our research center in Bronx County, NY. Written informed consent was obtained at the first study visit in accordance with study protocols approved by the institutional review board of the Albert Einstein College of Medicine. Participants completed demographic, psychosocial, neurological, and neuropsychological assessments as part of the EAS battery. A detailed description of this can be found in Katz et al. [17]. The PSS was added to the battery in 2006.

A detailed description of the determination of clinical outcomes can be found in Katz et al. [17]. Participants were assigned a diagnosis of aMCI using the Petersen Criteria [18]. In brief, it required the presence of subjective memory complaints, objective memory impairment, and no current diagnosis of dementia. A diagnosis of dementia was made at case conference in accordance with the fourth edition of the Diagnostic and Statistical Manual of Mental Disorders [19].

### 2.3 Cognitive Domain Evaluation

In a previous study, we used principal component analysis (PCA) to identify domains of cognitive performance in this sample [20]. A more detailed description can be found elsewhere [20]. In summary, three cognitive domain components emerged from the PCA: a language component, an episodic memory component, and a frontal-executive component.

The language domain was evaluated using the Boston Naming Task (BNT), Wechsler Adult Intelligence Scale III (WAIS-III) vocabulary test, and Logical Memory I subtest from the Wechsler Memory Scale-Revised (WMS-R). The BNT [21] is a measure of confrontational naming where the participant names pictures of objects ranging from common to rare. Scores range from 0–60 with a higher score corresponding to better function. The WAIS-III Vocabulary test [22] is an index of verbal comprehension. The participant is asked to define 33 words. Scores range from 0–66. The Logical Memory I subtest of the WMS-R [23] is a test of immediate declarative memory. Two stories are read to the participant, and after each story, the participant is asked to recall it from memory. The scores range from 0–50.

The episodic memory domain was evaluated using the Free and Cued Selective Reminding Test (FCSRT) and category fluency test. The FCSRT [24,25] is an episodic memory test where the participant learns 16 pictures by identifying and naming each one. There are 3 trials of immediate free recall, each of which is followed by category cues for the items not freely recalled. The total score ranges from 0–48. Category fluency [26] is assessed by asking the participant to name as many words that belong to a particular category (animals, fruits, vegetables) in one minute. The score is the total number of correctly named items.

The frontal-executive domain was evaluated using the WAIS-III Block Design test, WAIS-III Digit Symbol test, and Trail Making Test part A and B. The WAIS-III Block Design test [22] measures visuospatial and abstract reasoning. The participant is shown a set of 14 printed geometric patterns and asked to replicate them using two-color blocks. The scores range from 0–68. The WAIS-III Digit Symbol test [22] assesses processing speed. The participant copies symbols under the corresponding numbers using a key at the top of the page. The number of correctly drawn symbols in 120 seconds determines the score (range 0–133). In the Trail Making Test [27] part A (TMTA), the participant is given a sheet with the numbers 1–25 placed inside circles and the participant has to connect the numbers in order as quickly as possible. In the Trail Making Test part B (TMTB), the sheet has both numbers (1–13) and letters (A-M) placed inside circles. The participant has to connect the numbers and letters in alternating sequences in order as quickly as possible. The score for both TMTA and TMTB is the seconds to completion.

### 2.4 Evaluating Perceived Stress

The PSS [12] is a 14 item questionnaire that assesses perceived stress in the past four weeks. Each item is rated on a five point Likert-like scale (0 = never to 4 = very often). Six of the fourteen items have negative content (1, 2, 3, 8, 11, 14) and the remaining seven have positive content (4, 5, 6, 7, 9, 10, 13). An example of a positively worded item is “How often have you felt that you were effectively coping with important changes that were occurring in your life?”. An example of a negatively worded item is “How often have you felt difficulties were piling up so high that you could not overcome them?”. Item 12 (“how often have you found yourself thinking about things that you have to accomplish?”) did not load well on either factor and is considered neither positive nor negatively worded. The positively worded subscale (PSS-PW) score was calculated after reversing positively worded items’ scores and then summing all scores; PSS-PW scores ranged from 0 to 28. The negatively worded subscale (PSS-NW) score was calculated by summing the score for the negatively worded items, with the exception of item 12; PSS-NW scores ranged from 0 to 24. Internal consistency was good for the PSS-PW (α = 0.84) and adequate for the PSS-NW (α = 0.78).

### 2.5 Depression

The Geriatric Depression Scale (GDS) was used to assess depressive symptoms [28]. The GDS is a 15-item scale in which participants respond with either “yes” or “no” to questions about how they felt over the past week. Scores range from 0–15, with higher scores indicating greater depressive symptomatology. A score of 6 or greater was classified as having depressive symptoms [29]; this value was found to have high sensitivity (0.97) and specificity (0.96) in a study of Japanese older adults. The GDS has both good reliability (α=0.80) [30] and was associated with depression as defined by the International Classification of Diseases and DSM-IV [29].

### 2.6 Statistical Analyses

To reduce multiple comparisons, we applied the gatekeeper approach in all analyses [31]. We first assessed the bivariate Pearson’s correlation between the PSS-14, PSS-PW, and PSS-NW and each of the three cognitive domain component. If the correlation between the PSS and cognitive domain was significant, we planned to use bivariate Pearson’s correlations to examine the unadjusted relationships between the PSS and each individual cognitive tests making up each cognitive domain. If the correlation between the PSS and the cognitive domain was not significant, we did not test relationships between the PSS and each individual test within the cognitive domain.

Finally, we examined the adjusted relationships between the PSS and individual cognitive tests using a series of regression analyses. First, we used linear regression analyses to examine relationships between individual cognitive tests (outcomes) and the PSS-PW, PSS-NW, and PSS-14, adjusting for age, sex, and race. Then, we further adjusted these models for years of education and depressive symptoms. Standardized betas were reported instead of coefficients to better allow comparison across analyses. All analyses were performed using STATA, version 12.1 (College Station, Texas). No statistical power calculation was performed. Instead, all available participants of the EAS who had PSS scores and cognitive evaluations performed on the same year were included in the study sample.

## 3. Results

Table 1 shows the characteristics of the study sample. In summary, the average age of the participants was 79.1 and 563 (62.8%) are female. The population is 63.5% (n = 570) white and 29.0% (n = 260) black, with the remainder in other racial groups (n = 67).

Table 2 shows the bivariate associations between the two subscales of the PSS, PSS-14, and the cognitive domain components. The increased PSS-PW scores are significantly associated with decreased cognitive domain scores for language (r = −0.15, p < 0.001), episodic memory (r = −0.10, p = 0.002), and frontal-executive functioning (r = −0.21, p <0.001). Increased PSS-14 scores are also significantly associated with decreased cognitive domain scores for language (r = −0.09, p = 0.011), episodic memory (r = −0.10, p = 0.002), and frontal-executive functioning (r = −0.10, p = 0.001). The PSS-PW is also significantly associated with all the cognitive tests comprising each cognitive domain component. The PSS-NW is not significantly associated with any domain score including language (r = 0.07, p = 0.051), episodic memory (r = 0.01, p = 0.686), nor frontal-executive functioning (r = 0.03, p = 0.580). Applying the gatekeeper approach, we did not examine the association between the PSS-NW and the individual cognitive tests.

Table 3 shows linear regressions with outcomes of each cognitive domain component and individual cognitive tests, and predictors of interest being PSS-PW, PSS-NW, and PSS-14. These models are adjusted for age, white race, and gender. Increased PSS-PW is associated with lower language function scores (β = −0.19, p < 0.001), lower episodic memory scores (β = −0.13, p < 0.001), and lower frontal-executive scores (β = −0.15, p < 0.001). Higher PSS-PW is also significantly associated with all the individual cognitive tests making up each component. The PSS-NW is not significantly associated with any of the cognitive domain components. Higher PSS-14 scores are associated with lower language function scores (β = −0.11, p = 0.001), lower episodic memory scores (β = −0.11, p = 0.001), and lower frontal-executive scores (β = −0.10, p = 0.001). Higher PSS-14 scores are associated with all the individual cognitive tests making up each component with the exception of LM (p = 0.068). In Table 4, we further adjust for years of education. Higher PSS-PW scores remain significantly associated with lower language cognitive functioning (β = −0.12, p < 0.001), episodic memory (β = −0.08, p = 0.015), and frontal-executive functioning (β = −0.07, p = 0.019). The PSS-PW remains significantly associated with the BNT, WAIS-III Vocabulary test, FCSRT, TMTA, TMTB, and WAIS-III Block Design. The PSS-NW remains unassociated with any of the cognitive domain components. The PSS-14 remains significantly associated with language (β = −0.09, p = 0.004) and episodic memory (β = −0.08, p = 0.014). However the association between the PSS-14 and frontal-executive function lost its significance (β = −0.05, p = 0.068). The PSS-14 remains significantly associated with the BNT, WAIS Vocabulary test, and FCSRT.

## 4. Discussion

The current study examined cross-sectional relationships between the PSS-14 and its two subscales (PSS-PW and PSS-NW) and three domains of cognitive functioning (Language, Episodic Memory, and Executive Functioning). We found that increases in PSS-PW scores (which indicates lower endorsement of positive items on the PSS) were associated with reduced performance on the language component, episodic memory component, and frontal-executive component. In contrast, PSS-NW scores were not significantly correlated with any of these cognitive domain components. Higher PSS-14 scores were associated with reduced performance on the language component and the episodic memory component, but not the frontal-executive component after adjustment.

To the best of our knowledge, this is the first study that examines the relationship between PSS subscales and performance on a range of cognitive tests in nondemented older adults. Our results are consistent with previous studies using the PSS as a single summation score. Aggarwal et al. found that increased PSS scores were related to worse cognitive function in older adults [9]. There are several cross-sectional studies that have found an association between higher PSS total scores and memory decline. Potter et al. found that higher PSS scores, but not life events, were associated with more self-reported memory complaints in older women [32]. A study of HIV-infected women found that the top tertile of PSS scores correlated with worse verbal learning and memory [33]. Another study found that higher PSS scores were associated with worse memory and executive function in college students [34]. VonDras et al. found that higher PSS scores were associated with worse episodic memory test scores in young, midlife, and older adults, particularly those that required greater executive resources [35]. These studies, in combination with our current results, provide further support for the association between stress and cognitive decline.

We found that the PSS-PW was better associated with cognitive function than the PSS-14. We believe that this is because the association between the PSS-14 and cognitive function has been diluted by incorporating the PSS-NW scores. We found that the PSS-NW scores were not correlated with any cognitive function measure. Our results also add to the growing literature that the subscales of the PSS may measure different constructs of stress [14,15,36]. In the current study, we found that the PSS-PW, but not the PSS-NW, was associated with language, episodic memory, and frontal-executive function. This is consistent with previous studies in which the PSS-PW, but not the PSS-NW, predicted transitions from normal cognition to aMCI and from aMCI to dementia in a community sample of older adults [11,16]. Other studies have also found the two subscales to have differential properties [14,15,36]. Hewitt et al. found that the PSS-NW, but not the PSS-PW, was correlated with depression in an adult psychiatric sample [14]. Sanders et al. found that only the PSS-PW predicted tooth loss [36].

The transactional model of stress suggests that stress occurs when an individual perceives coping resources to be insufficient to handle a stressor [37]. It has been suggested that the PSS-PW may measure coping ability and the PSS-NW may measure stressor severity (distress) [14]. This would be consistent with the current results since one would expect that someone who is better able to cope with stress may be able to do so due to better executive function, memory, and general cognitive ability. Someone who is high functioning may have many things going on in their life, and may have more events that could cause stress. So the amount of distress or stressful events would not be expected to correlate with cognitive function. However, we call the subscales PSS-PW and PSS-NW to avoid prematurely characterizing the subscales. Future research should examine the construct validity of the two subscales by comparing them to tests measuring coping and distress. This will improve the ability of researchers to interpret differential associations between the PSS-PW and PSS-NW in a variety of contexts, including cognitive functioning.

Future research should also study how the PSS subscales relate to biological markers of stress such as salivary cortisol secretion. This will improve our ability to elucidate the mechanisms of the role of stress in cognitive functioning. For example, chronic stress can increases the glucocorticoid release. Glucocorticoids increase the vulnerability of the brain to metabolic insults [38], which can cause the impairment of cognitive function we see here. However, we do not know how the PSS subscales differentially influence biological mechanisms of stress, limiting our ability to draw mechanistic inferences.

The major limitation of this study is its cross sectional nature. Therefore it is impossible to determine causality or temporal sequence. We tested the robustness of our findings by using PSS-PW and PSS-NW scores from one year prior to the cognitive test scores and found similar results. Thus, the relationship between PSS-PW and cognitive test scores is apparent when cognitive scores are assessed concurrently, and one year in the future. It is unlikely to be due to a change in PSS scores over time since we have found that PSS total, PSS-PW, and PSS-NW scores are relatively constant over time [11]. This suggests that PSS and PSS subscales are more likely to be measuring traits rather than states. Another limitation of the study is that the tests used to assess the frontal-executive domain are limited in scope. These tests used primarily tested aspects of reasoning and problem solving, but did not assess planning, language, long-term memory, impulse control, emotions, motor function, or behavior. Future studies could use the Stroop effect as a measure of frontal-executive function.

The PSS-PW was significantly associated with all of the cognitive tests in univariate tests; however, the correlations had very small magnitudes. While the magnitudes of the correlations were small, we find it interesting that the results were very consistent. In these analyses, there was no adjustment for multiple comparisons or for other possible confounding factors. We used the gatekeeper method to reduce the possibility of capitalizing on chance and limiting the number of analyses. While we did not further correct for multiple comparisons, we believe that the consistency of our results makes it unlikely so see only the PSS-PW correlating with so many of the cognitive tests while the PSS-NW does not based solely on chance.

In adjusted analyses, we found that the PSS-PW was associated with BNT, WAIS-III Vocabulary test, FCSRT, TMTA, TMTB, and WAIS-III Block Design. Adjusting for the education and depressive symptoms attenuated the relationship between the PSS-PW and LM, Category Fluency, and WAIS-III Digit Symbol. It also attenuated the relationship between the PSS-14 and executive function, and Category Fluency. This suggests that these tests are very strongly correlated with education and depressive symptoms. Surprisingly, the PSS-PW was associated with all three cognitive domains. One possible explanation for this is that we did not test enough cognitive domains. Alternatively, it is possible that there is that education and depressive symptoms are confounders between cognitive ability and PSS-PW score. Future studies should investigate the relationship between the PSS-PW and education to determine if the PSS-PW acts as a measure of cognitive reserve.

The EAS population is relatively well educated with an average of 14.3 years of education. It is well known that education level is highly associated with some of the cognitive tests [39]. We did not find an interaction between neither the PSS-PW nor PSS-NW and education. Future research should investigate whether the current findings still apply in older adults with less education.

There are a number of strengths in this study. The EAS population was recruited systematically from Bronx County, NY and is ethnically diverse. There are well established and validated procedures for establishing cognitive status and the EAS cognitive battery is extensive. The PSS has been validated for use in older adults using the EAS population [13].

This study adds to the literature of the associations between high PSS scores and adverse effects of stress. Future studies should examine the mechanism of how the PSS-PW is related to cognitive domain functions. Subscale-level analysis provides a higher level of specificity than scale-level analysis, and future research should evaluate the physiologic, cognitive and emotional components of stress associated specifically with the PSS-PW (as opposed to the PSS-NW) to better differentiate between the stress construct measured by each subscale. It is possible that certain stress management interventions could target PSS-PW specifically in order to reduce cognitive decline. Further research should evaluate the effects of stress reduction interventions on the PSS as subscales, instead of as a single summation score.

## Figures and Tables

**Table 1:** Characteristics of the study population (n = 897)

Variable	Mean (SD)
Age	79.1 (5.3)
Female, n (%)	563 (62.8)
Race	
%White, n (%)	570 (63.5)
%Black, n (%)	260 (29.0)
%Other, n (%)	67 (7.5)
Years of Education	14.3 (3.4)
GDS	2.0 (2.1)
PSS-PW	9.3 (5.3)
PSS-NW	7.0 (4.4)
Time Enrolled in Study	3.2 (2.9)
BNT	12.1 (2.4)
WAIS-III Vocabulary	46.2 (12.2)
LM	21.3 (6.7)
FCSRT	32.1 (5.3)
Category Fluency	38.1 (9.3)
TMTA	0.02 (0.01)
TMTB	0.01 (0.004)
WAIS-III Digit Symbol	46.4 (14.0)
WAIS-III Block Design	24.3 (8.9)

GDS, Geriatric Depression Scale; PSS-PW, positively worded subscale score of the Perceived Stress Scale-14 (PSS); PSS-NW, negatively worded subscale score of PSS; BNT, Boston Naming Task; WAIS-III, Wechsler Adult Intelligence Scale III; LM, Logical Memory I subtest from the Wechsler Memory Scale-Revised; FCSRT, Free and Cued Selective Reminding Test; TMTA, Trail Making Test part A, TMTB, Trail Making Test part B

**Table 2:** Bivariate Cross-sectional Associations of PSS-PW and PSS-NW with Neurocognitive Performance

Neurocognitive Performance	PSS-PW		PSS-NW		PSS-14	

	Corr	P value	Corr	P value	Corr	P value
**Language function**	−**0.15**	**<0.001**	0.07	0.051	−**0.09**	**0.011**
BNT	−**0.19**	**<0.001**			−**0.09**	**0.006**
WAIS-III Vocabulary	−**0.22**	**<0.001**			−**0.05**	**0.106**
LM	−**0.14**	**0.003**			−0.06	0.089
**Episodic memory function**	−**0.16**	**<0.001**	0.01	0.686	−**0.10**	**0.002**
FCSRT	−**0.14**	**0.002**			−**0.09**	**0.010**
Category Fluency	−**0.13**	**0.004**			−**0.09**	**0.012**
**Frontal-executive function**	−**0.21**	**<0.001**	0.03	0.580	−**0.09**	**0.007**
TMTA	−**0.17**	**<0.001**			−**0.10**	**0.004**
TMTB	−**0.21**	**<0.001**			−**0.07**	**0.032**
WAIS-III Digit Symbol	−**0.16**	**<0.001**			−0.06	0.090
WAIS-III Block Design	−**0.14**	**0.002**			−**0.11**	**0.001**

PSS-PW, positively worded subscale score of the Perceived Stress Scale-14 (PSS); PSS-NW, negatively worded sub-scale score of PSS; Language PCA is made up of BNT, WAIS-III Vocabulary, LM; BNT, Boston Naming Task; WAIS-III, Wechsler Adult Intelligence Scale III; LM, Logical Memory I subtest from the Wechsler Memory Scale-Revised; Memory PCA is made up of FCSRT and Category Fluency; FCSRT, Free and Cued Selective Reminding Test; Executive PCA is made up of WAIS-III Block Design, WAIS-III Digit Symbol, TMTA, TMTB; TMTA, Trail Making Test part A, TMTB, Trail Making Test part B

**Table 3:** Cross Sectional Relationships between PSS-PW, PSS-NW, and Neuropsychological Performance Adjusted for Age, White Race, and Gender

Neuropsychological Performance	PSS-PW		PSS-NW		PSS-14	

	Beta	P value	Beta	P value	Beta	P value
**Language function**	−**0.19**	**<0.001**	0.00	0.995	−**0.11**	**0.001**
BNT	−**0.14**	**<0.001**			−**0.10**	**0.002**
WAIS-III Vocabulary	−**0.18**	**<0.001**			−**0.08**	**0.010**
LM	−**0.10**	**0.004**			−0.06	0.068
**Episodic memory function**	−**0.13**	**<0.001**	−0.06	0.071	−**0.11**	**0.001**
FCSRT	−**0.09**	**0.005**			−**0.08**	**0.016**
Category Fluency	−**0.10**	**0.001**			−**0.10**	**0.003**
**Frontal-executive function**	−**0.15**	**<0.001**	−0.03	0.684	−**0.10**	**0.001**
TMTA	−**0.13**	**<0.001**			−**0.09**	**0.007**
TMTB	−**0.12**	**<0.001**			−**0.07**	**0.020**
WAIS-III Digit Symbol	−**0.11**	**<0.001**			−**0.07**	**0.025**
WAIS-III Block Design	−**0.12**	**<0.001**			−**0.12**	**<0.001**

* PSS-PW, positively worded subscale score of the Perceived Stress Scale-14 (PSS); PSS-NW, negatively worded sub-scale score of PSS; Language PCA is made up of BNT, WAIS-III Vocabulary, LM; BNT, Boston Naming Task; WAIS-III, Wechsler Adult Intelligence Scale III; LM, Logical Memory I subtest from the Wechsler Memory Scale-Revised; Memory PCA is made up of FCSRT and Category Fluency; FCSRT, Free and Cued Selective Reminding Test; Executive PCA is made up of WAIS-III Block Design, WAIS-III Digit Symbol, TMTA, TMTB; TMTA, Trail Making Test part A, TMTB, Trail Making Test part B

* The gatekeeper method was applied and p values were not calculated if there was no association found with overall cognitive domain function.

**Table 4:** Cross Sectional Relationships between PSS-PW, PSS-NW, and Neuropsychological Performance Further Adjusted for Years of Education and Depression

Neuropsychological Performance	PSS-PW		PSS-NW		PSS-14	

	Beta	P value	Beta	P value	Beta	P value
**Language function**	−**0.12**	**<0.001**	−0.00	0.462	−**0.09**	**0.004**
BNT	−**0.09**	**0.004**			−**0.08**	**0.011**
WAIS-III Vocabulary	−**0.11**	**<0.001**			−**0.06**	**0.028**
LM	−0.04	0.183			−0.04	0.202
**Episodic memory function**	−**0.08**	**0.015**	−0.06	0.088	−**0.08**	**0.014**
FCSRT	−**0.07**	**0.044**			−**0.07**	**0.023**
Category Fluency	−0.06	0.070			−0.06	0.100
**Frontal-executive function**	−**0.07**	**0.019**	−0.02	0.451	−0.05	0.068
TMTA	−**0.09**	**0.010**				
TMTB	−**0.06**	**0.043**				
WAIS-III Digit Symbol	−0.05	0.128				
WAIS-III Block Design	−**0.07**	**0.037**				

* Depression (as measured by the Geriatric Depression Scale dichotomized at 6)

* PSS-PW, positively worded subscale score of the Perceived Stress Scale-14 (PSS); PSS-NW, negatively worded sub-scale score of PSS; Language PCA is made up of BNT, WAIS-III Vocabulary, LM; BNT, Boston Naming Task; WAIS-III, Wechsler Adult Intelligence Scale III; LM, Logical Memory I subtest from the Wechsler Memory Scale-Revised; Memory PCA is made up of FCSRT and Category Fluency; FCSRT, Free and Cued Selective Reminding Test; Executive PCA is made up of WAIS-III Block Design, WAIS-III Digit Symbol, TMTA, TMTB; TMTA, Trail Making Test part A, TMTB, Trail Making Test part B

* The gatekeeper method was applied and p values were not calculated if there was no association found with overall cognitive domain function.
